# Lymphopenia and IgG2 subclass deficiency in patients with severe COVID-19 pneumonia 

**DOI:** 10.7196/AJTCCM.2021.v27i2.134

**Published:** 2021-03-30

**Authors:** E M Taban, G R Tintinger, D Joseph, P Gaylard, G Richards

**Affiliations:** 1 Mediclinic Midstream Hospital, Johannesburg, South Africa; 2 Department of Internal Medicine, Steve Biko Academic Hospital and Faculty of Health Sciences, University of Pretoria, Pretoria, South Africa; 3 Data Management and Statistical Analysis, Johannesburg, South Africa; 4 Department of Critical Care, Faculty of Health Sciences, University of the Witwatersrand, Johannesburg, South Africa

**Keywords:** IgG2 subclass deficiency, COVID-19

## Abstract

**Background:**

COVID-19 caused by the novel severe acute respiratory syndrome coronavirus-2 (SARS-CoV-2) manifests with a range of disease severities. A small proportion of COVID-19 patients are severely ill; however, a significant proportion of these patients are critically ill, and require admission and mechanical ventilation, which is associated with a high mortality.

**Objective:**

To identify factors that may predispose patients with COVID-19 to severe disease that requires mechanical ventilation (MV).

**Methods:**

We performed a retrospective audit of patients admitted with COVID-19 pneumonia to the intensive care unit (ICU) and medical wards to evaluate the potential associations between comorbid conditions, lymphopenia and IgG subclass deficiency with a need for MV.

**Results:**

A total of 51 patients were included in the study. Almost half of the patients (47%; n=24) were documented to have IgG2 deficiency, 43% (n=22) had lymphopenia and 37% (n=19) had combined lymphopenia and IgG2 subclass deficiency. Of the 24 patients who required MV, 75% had IgG2 subclass deficiency, 73% had lymphopenia and 50% had both. The relative risk for requiring MV was 2.64, 3.38 and 2.81 for lymphopenia, IgG2 subclass deficiency and both, respectively.

**Conclusion:**

These findings suggest that lymphopenia, low IgG2 concentrations or the combination of both may be used to identify patients with severe COVID-19 that are at increased risk for MV. This may facilitate earlier identification of patients at high risk, who may benefit from more intensive therapy.

## Background

Severe acute respiratory syndrome coronavirus-2 (SARS-CoV-2) is the novel coronavirus that causes COVID-19.^[1]^ At the time of writing this manuscript (5 September 2020) and since its initial detection, more than 41 million cases have been confirmed and 1 139 420 deaths have occurred worldwide. Similarly, the number of cases in South Africa (SA) has continued to rise and it is currently reported that there are 708 359 confirmed cases and 18 741 deaths, since the first cases were diagnosed in March 2020.^[2]^

The spectrum of disease is highly variable, with 80.9% of patients with COVID-19 remaining asymptomatic, 13.9% progressing to severe disease and 4.7% become critically ill.^[3]^ Of the critically-ill patients admitted to an intensive care unit (ICU), more than 50% will ultimately require mechanical ventilation (MV) with an estimated mortality rate of 50 - 80%.^[4]^ These patients frequently have a prolonged course in the ICU, which is often complicated by secondary hospital-acquired infections, which are associated with a high mortality.^[5]^

Human immunoglobulins (Igs) are comprised of five classes: IgG, IgM, IgE, IgD and IgA – each having a specific function. IgA has two subclasses and IgG has four subclasses, which are defined by the unique antigenic structures of their heavy chains.

The most common antibody found in the blood and extracellular fluid is IgG. Its function is to bind many types of pathogens such as viruses, bacteria and fungi, and protect the body from infection.^[6]^ IgG subclass proportions are tightly controlled within a narrow range: IgG1 (60 - 65%); IgG2 (20 - 25%); IgG3 (5 - 10%); and IgG4 (3 - 6%) of total IgG.

IgG subclass deficiency has been reported in patients with recurrent sinopulmonary infections^[7]^ and may be associated with an IgG1 deficiency.^[8]^

Patients with primary immunodeficiency and combined variable immunodeficiency have been treated with immunoglobulin replacement therapy since the licensing of intravenous Ig in 1980. Nguyen *et al*.^[9]^ recently reviewed the use of intravenous Ig and hyperimmune globulin therapy in the treatment of COVID-19 infections, where they postulated that these agents may act as potential modulators of inflammation.

It has also been postulated that during severe infection with coronaviruses, a class switch results in the body producing more IgG1 and IgG3.^[10]^ Previous studies have reported IgG2 subclass deficiency in patients with severe influenza (H1N1)^[11,12]^ but there are no studies to our knowledge that have evaluated IgG subclasses in patients admitted with COVID-19 pneumonia. The primary objective of the present study was to retrospectively probe the potential associations between comorbid conditions, lymphopenia, IgG2 subclass deficiency and disease severity, progression to MV or death in patients admitted with COVID-19 pneumonia.

## Methods

We conducted a retrospective audit of patients with COVID-19 pneumonia admitted to the ICU and medical wards at Mediclinic Midstream Hospital, Pretoria, SA, from 7 August to 7 September 2020. EMT obtained informed consent from patients or their relatives prior to inclusion in the study.

### Inclusion criteria 

All patients admitted with a confirmed positive SARS-CoV-2 PCR test result, had mild to severe COVID-19 pneumonia, and radiological evidence of COVID-19 pneumonia reported according to the British Society of Thoracic Imaging recommendations were included in the study.

**The following criteria were used to differentiate mild, moderate and severe acute respiratory distress syndrome (ARDS):**

- Mild ARDS: admission to a general ward with a partial pressure O_2_(PaO_2_)/fraction of inspired oxygen (FiO_2_) ratio >200.
- Moderate ARDS: ICU admission with a PaO_2_/FiO_2_ ratio of 101 - 200, required non-invasive ventilation or high-flow nasal oxygen.
- Severe ARDS: ICU admission with a PaO_2_/FiO_2_ ratio <100 and required MV.

### Exclusion criteria

All patients with underlying immunodeficiency or conditions known to cause IgG subclass deficiency including influenza infection were excluded.

### Measurements

Routine laboratory testing included full blood count (FBC) and serum Ig and IgG subclass levels. Lymphopenia was defined as a lymphocyte count <1.0 × 10^9^/L.

### Laboratory testing for IgG subclasses

The IgG subclasses were determined using latex-enhanced immunonephelometry using the Siemens BNP ProSpec System (Siemens, Germany) and total IgG, IgA and IgM were measured using the immunoturbidimetric method on the Abbott Architect 4100 Analyzer (Abbot, USA), while IgE was determined using enzyme-enhanced chemiluminescence on the Siemens IMMULITE 2000 immunoassay system (Siemens, Germany). The reference range for total IgG was 5.52 - 16.31 g/L, IgG1 (4.05 - 10.1 g/L), IgG2 (1.69 - 7.86 g/L), IgG3 (0.11 - 0.85 g/L) and IgG4 (0.03 - 2.01 g/L) in healthy adults according to the manufacturer’s specifications.

### Sample size estimation and statistical analyses

Based on a 44% prevalence of IgG2 subclass deficiency as a risk factor, 72% incidence of severe disease and an estimated relative risk (RR) of 1.5 or greater with 80% power at the 5% significance level, the minimum required sample size is 195. The actual sample size of about 50 patients allows for the detection of RR of 1.9 or greater, which is adequate for a study of this nature.^[13]^

The RR of each study variable for the outcomes (disease severity, IgG2 subclass deficiency, ventilation status and mortality) was determined together with the 95% confidence interval using binomial regression. Study variables significant at p<0.20 were combined into a multivariable model after examining each pair of variables for possible confounding using the χ² test or the Fisher’s exact test for 2 × 2 tables. Comparison between selected study variables, and ventilation status and mortality were made using the independent samples *t*-test (or the Wilcoxon rank sum test where the data do not meet the assumptions of the *t*-test). Data analysis was performed using SAS software (SAS Institute Inc., USA), version 9.4 for Windows. A 5% significance level was used.

## Results

A total of 51 adult patients were included in the study (31 males and 20 females), with a mean (standard deviation (SD)) age of 56.5 (13.3) years. The demographic characteristics as well as the comorbidities, treatment and outcomes are shown in Table 1.

**Table 1:** Data for age, BMI and comorbid conditions, as well as the treatment and outcome of the study population

**Characteristic**	***n* (%)**
**Age (years)**	
31 - 49	17 (33)
50 - 64	20 (39)
65 - 77	14 (28)
**BMI (kg/m²)**	
18 - 25	6 (12)
26 - 30	15 (29)
≥31	30 (59)
**Comorbidities**	
Hypertension	29 (57)
Diabetes mellitus	21 (41)
Hyperlipidaemia	13 (26)
HIV	4 (8)
Asthma/COPD	3 (6)
Ankylosing spondylitis	1 (2)
Atrial fibrillation	1 (2)
Colon cancer	1 (2)
Stroke	1 (2)
Hypothyroidism	1 (2)
Coronary artery disease	2 (4)
Congenital heart disease	1 (2)
Pulmonary hypertension	1 (2)
Pulmonary fibrosis	1 (2)
Chronic renal failure	2 (4)
**Number of comorbidities (grouped)**	
0 - 1	16 (31)
2 - 3	22 (43)
≥4	13 (25)
**Treatment**	
Intravenous immunoglobulin	17 (34)
High-flow nasal oxygen	27 (33)
Mechanical ventilation	24 (47)
**Outcome**	
Survivors	41 (80)
Non-survivors	10 (20)

BMI = body mass index

COPD = chronic obstructive pulmonary disease

Thirty patients had a BMI >30 kg/m² and the most common comorbid conditions were hypertension (57%), diabetes mellitus (41%), hyperlipidaemia (26%) and HIV (8%). About a quarter (24%; n=12) of patients were current smokers. The percentage of patients with 2 - 3 comorbidities was 43% (n=22) and those with 4 or more comorbidities was 25% (n=13). Six patients developed renal failure and three were diagnosed with cardiac failure. Intravenous Ig was administered to 34% (n=17) of patients and 47% (n=24) required intubation and MV

The mortality rate observed in the present study was 20% (n=10). The causes of death were septic shock (n=4), tracheostomy-related complications (n=2) and the remainder (n=4) died of cardiac failure, extradural haematoma, myocardial infarction and a pneumothorax.

The PaO_2_/FiO_2_ ratios, lymphocyte counts, and Ig subclasses of patients included in the study are shown in Table 2. Almost one-third (32%; n=16) of patients had severe disease, 30% (n=15) had moderate disease and 37% (n=19) had mild disease based on the criteria for ARDS described above. The median (IQR) PaO_2_/FiO_2_ ratio for all patients (excluding one with no data) was 165 (96 - 285).

**Table 2:** PaO_2_/FiO_2_ ratios, immunoglobulin subclasses (g/L) and lymphocyte counts (×10^9^/L) of the study population

**Characteristic**	***n*(%)**	Mean (SD)*
**PaO_2_/FiO_2_**		
<100	16 (32)	-
101 - 200	15 (30)	-
≥201	19 (37)	-
**IgG1**		
Low	9 (18)	3.40 (0.45)
Normal/high	42 (82)	6.67 (2.09)
**IgG2**		
Low	24 (47)	1.31 (0.28)
Normal/high	27 (53)	2.78 (0.83)
**IgG3**		
Low	2 (4)	0.09 (0.02)
Normal/high	49 (96)	0.43 (0.36)
**IgG4**		
Low	1 (2)	0.02 (0.0)
Normal/High	50 (98)	0.35 (0.35)
**Lymphocyte count**		
Low	22 (43)	0.58 (0.22)
**IgG2 and lymphocyte count**		
Low IgG2 and lymphopenia	19 (37)	1.30 (0.30) and 0.56 (0.23)

PaO_2_ = partial pressure of oxygen

FiO_2_ = fraction of inspired oxygen

Almost half (47%; n=24) of patients had IgG2 deficiency and low concentrations of IgG1, IgG3 and IgG4 were detected in 18% (n=9), 4% (n=2) and 2% (n=1) patients, respectively.

The median (IQR) lymphocyte count was 1.1 (0.6 - 1.6) × 10^9^ /L and 43% (n=22) of patients had a lymphopenia. More than one-third (37%; n=19) of patients had both lymphopenia and IgG2 deficiency. Of the 24 patients who were intubated and ventilated, 67% had lymphopenia, 75% had low IgG2 levels and 63% had both lymphopenia and IgG2 deficiency.

The univariate RR of each study variable for disease severity, IgG2 subclass deficiency, ventilation status and mortality are shown in Table 3. Age (>50 years), hypertension, diabetes mellitus, 4 or more comorbidities, PaO_2_ /FiO_2_ ratio <200 and lymphopenia were all associated with IgG2 deficiency. Furthermore, age (50 - 64 years), hypertension, low IgG2 concentrations and the combination of lymphopenia and IgG2 deficiency were also all associated with severe ARDS (PaO_2_/FiO_2_ ratio <100).

**Table 3:** The relative risk of each study variable for the outcomes of IgG2 subclass deficiency, disease severity, requirement for mechanical ventilation and mortality

	**IgG2 subclass** **deficiency,** **RR (95% CI)**	**Severe disease,** **RR (95% CI)**	**Mechanical ventilation,** **RR (95% CI)**	**Mortality,** **RR (95% CI)**
**Gender**				
Female	1.00	1.00	1.00	1.00
Male	0.76 (0.43 - 1.35)	0.78 (0.51 - 1.20)	1.29 (0.68 - 2.44)	0.97 (0.31 - 3.01)
**Age**				
31 - 49	1.00	1.00	1.00	1.00
50 - 64	3.40* (1.15 - 10.09)	2.27* (1.15 - 4.47)	3.12* (1.04 - 9.37)	2.55 (0.29 - 22.31)
65 - 77	3.64* (1.21 - 10.93)	1.82 (0.86 - 3.87)	4.05* (1.38 - 11.91)	7.29 (0.99 - 53.58)
**BMI**				
18 - 25	-	-	-	-
23 - 30	1.00	1.00	1.00	1.00
≥31	1.14 (0.60 - 2.16)	0.77 (0.50 - 1.20)	0.88 (0.48 - 1.61)	1.75 (0.41 - 7.42)
**Comorbidities**				
Hypertension	2.28* (1.09 - 4.77)	1.85* (1.08 - 3.19)	1.84 (0.93 - 3.65)	3.03 (0.71 - 12.90)
Diabetes mellitus	2.38* (1.29 - 4.38)	1.34 (0.87 - 2.06)	2.00* (1.11 - 3.61)	3.33 (0.97 - 11.43)
Hyperlipidaemia	1.20 (0.65 - 2.23)	1.20 (0.76 - 1.88)	1.46 (0.83 - 2.58)	2.92* (1.00 - 8.50)
Smoking	1.34 (0.74 - 2.43)	0.95 (0.55 - 1.62)	1.08 (0.56 - 2.10)	1.39 (0.42 - 4.57)
**Number of** **comorbidities**				
0 - 1	1.00	1.00	1.00	1.00
2 - 3	2.00 (0.78 - 5.15)	1.18 (0.65 - 2.16)	1.60 (0.69 - 3.70)	2.91 (0.36 - 23.63)
≥4	2.77* (1.10 - 6.97)	1.54 (0.87 - 2.73)	1.97 (0.85 - 4.58)	6.15 (0.82 - 46.32)
**PaO_2_/FiO_2_ ratio**				
<100	3.27* (1.29 - 8.29)	-	3.09* (1.40 - 6.79)	2.97 (0.66 - 13.29)
101 - 200	2.85* (1.09 - 7.47)	-	1.52 (0.57 - 4.03)	1.90 (0.36 - 9.95)
≥201	1.00	-	1.00	1.00
**Lymphocytes**				
Lymphopenia	5.01* (2.22 - 11.31)	1.24 (0.80 - 1.91)	2.64* (1.39 - 5.01)	1.98 (0.63 - 6.17)
Normal	1.00	1.00	1.00	1.00
**IgG2**				
Low	-	2.05* (1.25 - 3.33)	3.38* (1.61 - 7.09)	2.63 (0.76 - 9.03)
Normal	-	1.00	1.00	1.00
**Lymphopenia** **+ low IgG2**				
Yes	-	1.58* (1.04 - 2.40)	2.81* (1.54 - 5.12)	2.53 (0.82 - 7.83)
No	-	1.00	1.00	1.00

RR = relative risk

CI = confidence interval

* p<0.05

Age (>50 years), diabetes mellitus, PaO_2_ /FiO_2_ <100, lymphopenia and IgG2 deficiency alone or in combination predicted progression to MV. Hyperlipidaemia was the only factor that was associated with mortality in this cohort of patients.

The study variables identified by means of univariate analysis were combined into a multivariable model. Lymphopenia was associated with IgG2 deficiency, age and hypertension were associated with more severe disease, and lymphopenia and IgG2 deficiency predicted the need for MV (Table 4).

**Table 4:** Multivariable analysis of factors associated with IgG2 deficiency, disease severity and the requirement for mechanical ventilation

	**Factors**	**RR (95% CI)**
Factors associated with IgG2 deficiency	Lymphopenia	5.01* (2.22 - 11.31)
Factors associated with disease severity	Age (50 - 64 years)	2.27* (1.15 - 4.47)
	Age (65 - 77 years)	1.82 (0.86 - 3.87)
	Hypertension	1.85* (1.08 - 3.19)
Factors associated with a requirement for mechanical ventilation^†^	Lymphopenia	2.64* (1.39 - 5.01)
	IgG2 deficiency	3.38* (1.61 - 7.09)
	Lymphopenia and IgG2 deficiency	2.81* (1.54 - 5.12)

RR = relative risk

CI = confidence interval

* p<0.05

^†^ These factors are confounded and were identified in separate multivariable models.

The comparison of patients who required MV with those who did not, and survivors with non-survivors is shown in Table 5. The PaO_2_ /FiO_2_ ratio, lymphocyte counts and IgG2 concentrations were significantly lower in patients who required MV. A similar, but insignificant, trend was observed in non-survivors v. survivors.

**Table 5:** Comparison of ventilated and non-ventilated patients as well as survivors and non-survivors

	Ventilated (n=24), median (IQR)*	Non-ventilated (n=27), median (IQR)*	***p*-value**
PaO_2_/FiO_2_	197 (61 - 183)	216 (133 - 309)	0.0009
Lymphocyte count (×10^9^/L)	0.8 (0.4 - 1.1)	1.5 (1.0 - 2.0)	0.0001
IgG1 (g/L), mean (SD)	5.9 (2.2)	6.3 (2.4)	0.52
IgG2 (g/L)	1.42 (1.23 - 1.67)	2.35 (1.74 - 2.79)	0.0011
			
	**Non-survivors (n=10)**	**Survivors (n=41)**	
PaO_2_/FiO_2_	96 (58 - 160)	188 (99 - 300)	0.057
Lymphocyte count (×10^9^/L)	0.9 (0.4 - 1.1)	1.1 (0.6 - 1.7)	0.23
IgG1 (g/L), mean (SD)	5.8 (2.4)	6.2 (2.3)	0.64
IgG2 (g/L)	1.5 (1.2 - 1.7)	2.2 (1.5 - 2.6)	0.14

IQR = interquartile range

PaO_2_ = partial pressure of oxygen

FiO_2_ = fraction of inspired oxygen

SD = standard deviation

*Unless otherwise specified

## Discussion

The global coronavirus pandemic has compelled physicians who care for patients infected with SARS-CoV-2 to critically evaluate therapeutic interventions and outcomes. Although relatively few individuals who contract SARS-CoV-2 become severely ill and require MV,^[3]^ the mortality rate for this group is high.^[4]^ In the present study, a range of clinical and laboratory variables that may predict the need for MV in patients with moderate/severe COVID-19 pneumonia were evaluated retrospectively.

Interestingly, many patients who were admitted with lymphopenia and/or low concentrations of IgG2 required intubation and ventilation. These associations were significant and may allow physicians to identify subgroups of COVID-19 patients who are at risk of disease progression.

Lymphopenia is a common finding in patients with a variety of viral infections, including COVID-19.^[14]^ However, the association of low IgG2 concentrations and severe COVID-19 pneumonia complicated by respiratory failure is a novel finding.

Different pathogens may induce variable IgG subclass responses.^[15]^ Viral infections induce an antibody response predominantly consisting of IgG1 and IgG3 while bacterial infections are associated with the induction of IgG2 and IgG4 subclasses.^[16]^ An apparent deficiency of IgG2 has been reported in patients infected with the H1N1 virus and this correlated with worsened outcome.^[17]^ A study from Australia^[11]^ also found evidence of an IgG2 subclass deficiency in patients with severe H1N1 infections and the IgG2 deficiency was considered to be the underling risk factor for severity. A similar study from Hong Kong^[17]^ observed low IgG2 concentrations in patients with severe H1N1 infections, but concluded that this was secondary to the viral infection and not a predisposing factor for severity. The authors of this study attributed the IgG2 deficiency to cytokine dysregulation, and in support of this contention, high viral loads have been associated with this phenomenon.^[18]^ IgG subclass isotype switching is driven by the prevailing cytokine milieu^[19]^ and dysregulation of these cytokines may alter the profile of Ig and IgG subclasses that are produced.

The findings of the present study suggest that lymphopenia, low IgG2 concentrations or a combination of both may be used to identify patients with severe COVID-19 pneumonia who will require MV. This is important, as the early identification of patients at high risk may allow for timely intervention with therapies that might improve outcome. These include antivirals such as remdesivir, which appears to be most valuable if administered early, and in so doing reduce the viral load and attenuate cytokine dysregulation.^[20]^ Later in the course of disease, when cytokine dysregulation may have already occurred, the administration of intravenous immunoglobulins^[21]^ or convalescent plasma may be an appropriate therapeutic intervention.^[22]^ Antimicrobial therapy directed against bacterial pathogens may also be useful in this setting, as low concentrations of IgG2 that develop during viral pneumonia increase the risk of secondary bacterial infections.^[17]^

Although the present study did not identify factors associated with mortality, it did show that lymphopenia and IgG2 subclass deficiency increase the risk of MV and predispose patients to worsened outcomes.

## Study limitations

Limitations of the study include the relatively small number of patients and the retrospective study design. Larger prospective studies evaluating the incidence of lymphopenia and IgG2 subclass deficiency and therapy with immunoglobulins in patients with severe COVID-19 pneumonia and the need for MV, may be useful.

## Conclusion

The high incidence of lymphopenia and IgG2 subclass deficiency in patients with severe COVID-19 pneumonia who required MV observed in this study may allow the institution of antiviral therapies or immunomodulatory drugs early to prevent disease progression.
